# Proangiogenic Features of Mesenchymal Stem Cells and Their Therapeutic Applications

**DOI:** 10.1155/2016/1314709

**Published:** 2016-01-06

**Authors:** Hongyan Tao, Zhibo Han, Zhong Chao Han, Zongjin Li

**Affiliations:** ^1^Department of Pathophysiology, Nankai University School of Medicine, Tianjin 300071, China; ^2^The Key Laboratory of Bioactive Materials, Ministry of Education, Nankai University College of Life Science, Tianjin 300071, China; ^3^State Key Lab of Experimental Hematology, Institute of Hematology and Blood Diseases Hospital, Chinese Academy of Medical Sciences, Tianjin 300020, China

## Abstract

Mesenchymal stem cells (MSCs) have shown their therapeutic potency for treatment of cardiovascular diseases owing to their low immunogenicity, ease of isolation and expansion, and multipotency. As multipotent progenitors, MSCs have revealed their ability to differentiate into various cell types and could promote endogenous angiogenesis via microenvironmental modulation. Studies on cardiovascular diseases have demonstrated that transplanted MSCs could engraft at the injured sites and differentiate into cardiomyocytes and endothelial cells as well. Accordingly, several clinical trials using MSCs have been performed and revealed that MSCs may improve relevant clinical parameters in patients with vascular diseases. To fully comprehend the characteristics of MSCs, understanding their intrinsic property and associated modulations in tuning their behaviors as well as functions is indispensable for future clinical translation of MSC therapy. This review will focus on recent progresses on endothelial differentiation and potential clinical application of MSCs, with emphasis on therapeutic angiogenesis for treatment of cardiovascular diseases.

## 1. Introduction

Cardiovascular diseases (CVDs) are one of the major causes of morbidity and mortality worldwide [1]. The idea of promoting neovascularization and improving perfusion of ischemic tissue via angiogenesis is promising for treatment of CVDs. Since ischemic diseases are primarily caused by endothelial dysfunction, the logic behind therapeutic angiogenesis is to promote spontaneous tissue reparation via endothelial cells (ECs) and growth factor administration [2]. However, due to the limitation of expanding efficiency and postnatal cell sources, angiogenic therapy with ECs is not available in most cases [2, 3]. With their tremendous differentiation potency, the application of mesenchymal stem cells (MSCs) has been suggested to be a valuable alternative source for treatment of ischemic diseases. Moreover, MSCs hold great promise for tissue regeneration and revascularization through stimulating the secretion of various cytokines responsible for proangiogenic and antiapoptotic effects [4]. Thus, elucidating the paracrine effects and endothelial differentiation of MSCs will not only enhance our understanding of vascular disease pathogenesis, but also improve our ability to facilitate endothelial differentiation of MSCs for regeneration purposes.

## 2. Isolation and Characterization of MSCs

MSCs were first described as stromal cells residing in the bone marrow of rats, which have the capacity to transform into fibroblast-like cells during the processes of tissue repair [5, 6]. Later in the 1970s, Friedenstein et al. demonstrated heterogeneous populations of adherent cells within bone marrow, which can replicate as undifferentiated cells and can differentiate into a variety of mesenchymal cells, including osteoblasts, chondrocytes, myocytes, and adipocytes [7, 8]. Moreover, these kinds of cells are widely distributed and can be isolated from adult tissues including bone marrow, adipose tissue, and peripheral blood or neonatal birth-associated tissues like Wharton's Jelly and placenta [9].

Bone-marrow-derived MSCs (BM-MSCs) can be isolated from bone marrow aspiration, which are among the most frequently used types in regenerative studies. However, the way to get BM-MSCs is accompanied by a risk of infection and is painful for patients, which means that finding alternatives is essential in clinical practice. Fortunately, other than bone marrow, several kinds of MSCs have been isolated successfully. For example, peripheral blood-derived MSCs (PB-MSCs) can be isolated from mononuclear cells of peripheral blood [10]. Adipose-derived MSCs are usually obtained from adipose samples by enzymatic digestion [11]. Moreover, Wharton's Jelly of umbilical cord and placenta are considered to be convenient and readily alternatives to bone marrow [12, 13]. MSCs derived from Wharton's Jelly have the weakest expression of histocompatibility complex class I genes [14] and immune-related genes [15] while they do not express major histocompatibility complex class II genes [14], which means that Wharton's Jelly-derived MSCs are promising source for tissue regeneration in clinical application.

MSCs are partly differentiated progenitor cells containing multilineage stem cells, and there are no specific and reliable markers for defining native MSCs. Isolation and purification of MSCs usually involve density gradient centrifugation or immunophenotyping [16]. The phenotype of MSCs is determined by certain surface markers including CD49a (alpha-1 integrin), CD73 (ecto-5′-nucleotidase), CD105 (endoglin), MSC antigen-1, CD271 (adapalene), CD29, CD44, CD90, and CD106 (vascular cell adhesion molecule-1), but lack of CD34, CD45, CD14, HLA-DR, CD19, and CD79 [17, 18]. However, no specific single marker can be used to identify MSCs from other kinds of cell types. The lack of specific MSCs markers has thwarted the attempts to categorize these kind of stem cells* in vivo* [19]. The specific genotype and proteonomic profiles' analysis of multipotential MSCs clones has been carried out to further elucidate the characterization of MSCs which are promising to understand the mechanisms for maintaining or regulating those cells from different sources [20]. CD106 is mainly expressed on blood vessel endothelium, which also is an important marker for MSCs [13]. Moreover, MSCs with high expression of transmembrane protein cadherin-2 (N-cadherin) have revealed a higher probability to differentiate into cardiomyocytes, which indicates that MSCs can improve heart function directly [21]. The unique subpopulation of MSCs possessing specific differentiation potency may contribute to designed therapeutic strategies.

## 3. Therapeutic Application of MSCs for Vascular Regeneration

**Figure 1:**
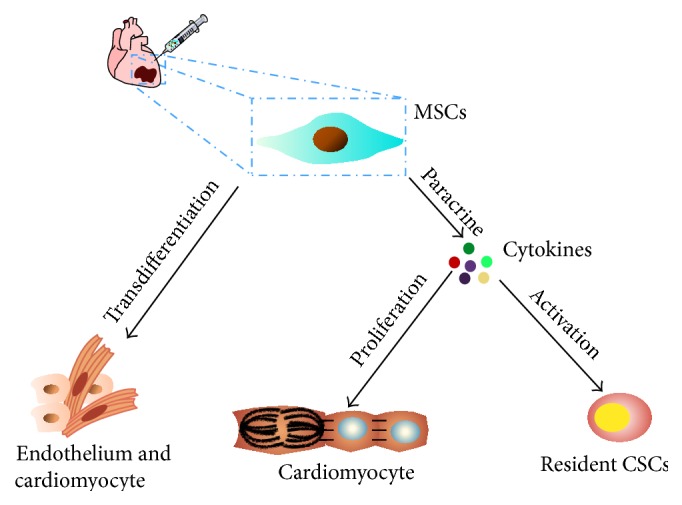
MSCs mediated therapy for myocardial infarction (MI). MSCs therapy could enhance heart function by (1) transdifferentiation into cardiomyocytes or endothelial cells (ECs) to replace the damage tissue and promote angiogenesis, respectively, (2) releasing soluble autocrine/paracrine factors, thereby activating endogenous adult cells involved in cells renewal/protection and neovascularization, and (3) stimulating endogenous resident cardiac stem cells (CSCs) proliferation and differentiation by paracrine soluble factors.

Despite advances in medical treatment, cardiovascular diseases (CVDs) are still major causes of adult death. Stem cell-based therapy in the treatment of ischemic diseases is a fast-growing field that has been proven effective and safe [22]. MSCs can transdifferentiate into all cell lineages of three germ layers including blood vessel cells arising from mesodermal tissue, which means that it is an attractive cell type for stem cell-based therapy for treatment of CVDs [23]. The therapeutic contribution of MSCs to tissue repair includes direct differentiation into injured cells including cardiomyocytes, smooth muscle cell, and ECs; presenting cytokines in the microenvironment; and stimulating endogenous stem cell differentiation (Figure 1). The most necessary property of MSCs for treatment of ischemic diseases is their differentiation potential toward vascular phenotypes, which can be identified by specific markers or functional assays.

### 3.1. The Application of MSCs for Ischemia Heart Disease Therapy

Among the various forms of ischemic diseases, ischemic heart disease is a serious disease caused by the imbalance between myocardial oxygen supply and demand [24, 25]. MSCs transplantation could promote myocardium repair by stimulating angiogenesis, and MSCs have emerged as a promising cell type for ischemic heart disease therapy both in small and large animal models [26]. For example, the intravenously injected MSCs derived from rat fetal heart for treatment of myocardial ischemia has achieved significant improvement in cardiac function [27]. The injected cells were found to express ECs and smooth muscle cells (SMCs) markers, suggesting that MSCs can transdifferentiate into SMCs and ECs. This result was also confirmed in large animal study. In a swine model of chronic ischemic cardiomyopathy, the engrafted MSCs were found to differentiate into cardiomyocytes, SMCs, and endothelium [28]. Moreover, the efficacy of intramyocardial injection of autologous MSCs has been confirmed in a clinical trial [29]. Here, we summarized the use of MSCs in clinical trials in CVDs treatment according to https://clinicaltrials.gov/ (Table 1). A number of trials are focused on the safety and efficacy of autologous/allogeneic MSCs transplantation. The results of these trials have confirmed that MSCs injection is safe and has the capability to improve cardiac function. Despite these positive results, additional clinical trials are still encouraged to determine whether stem cell-based therapy could serve as a novel alternative tool for treatment of ischemic heart disease.

Tissue injury and/or inflammation caused by ischemia can enhance the efficacy of MSCs homing to the injury tissue, which may provide an avenue for clinical translation of MSCs in the future as a cellular tool for CVDs therapeutics [30]. MSCs expressing various surface receptors could respond to the migratory signals released by sites of injury. Meanwhile, many studies to modify and/or enhance expression of these surface markers have been explored [31]. Therefore, optimized culture conditions, biomaterials, and gene transfection can be used to enhance migration ability of MSCs homing to the sites of injury. Although MSCs are the most promising sources of regeneration medicine, their poor cell viability after transplantation imposes restrictions on their clinical applications. Therefore, another aspect of research interest is improving cell survival rate after transplantation. MSCs genetically transduced with Akt1 (protein kinase B) can improve MSCs survival while infarcted myocardium reparation was enhanced [32]. The other approaches to enhance MSCs survival in ischemic tissue include hypoxic preconditioning, genetic modification, and angiogenin expression [33, 34], and these methods highlight the utility of gene-engineered cells and preconditioning cells.

The exact pathway of acute donor cell death following transplantation is still unknown, but lack of matrix support is one of major causes [2]. Engineered microenvironments with extracellular matrix and growth factors, mimicking the establishment of cell niche* in vivo*, have achieved great improvement in controlling stem cell fate [35–37]. Furthermore, the use of different polymers can help delivering drugs, viruses, plasmids, and cytokines [38]. Transplantation of the construct of tissue-engineered cardiac patch with BM-MSCs for immunocompetent rats after myocardial infarction has resulted in increased fractional shortening, augmented anterior wall thickness, and reduced left ventricle interior diameter at systole [39]. A similar study of a three-dimensional scaffolds transplantation that contain more than a few layers of BM-MSCs also led to heart function improvement and formation of MSC-derived neovasculatures [40].

### 3.2. The Application of MSCs for Treatment of Peripheral Artery Disease (PAD)

Peripheral artery disease (PAD) is a serious disease usually caused by atherosclerotic occlusion, which can lead to critical limb ischemia, tissue injuries, and eventual limb loss [41, 42]. The patients with PAD have a very poor long-term prognosis; only 30% of patients can live for 15 years after being diagnosed with this disease [43]. Critical limb ischemia is the most serious complication of PAD, which can cause pain, on walking or even at rest, and nonhealing ulcers. Although patients with limb ischemia are treated with a combination of methods, such as statins, antiplatelet drugs, and angioplasty, these treatments are occasionally insufficient to recover sufficient arterial blood flow to maintain tissue viability [44]. Therefore, it is urgent need to develop novel treatment approaches for revascularization of ischemic limbs to repair the injured tissues.

Currently, one of the most fundamental methods for PAD treatment is therapeutic angiogenesis, which aims to promote neovascularization and restore blood flow to ischemic limbs by forming new blood vessels from preexisting ones [45]. The evidences that MSCs transplantation could achieve this purpose have been confirmed by small and large animal studies [46, 47]. And the use of MSCs has already started in the first clinical trials and the safety/efficacy of MSCs was confirmed [48, 49]. In a double-blind, controlled, randomized trial in patients with critical limb ischemia caused by diabetes [50], ulcer size, pain-free walking time, and percutaneous tissue oxygen pressure were significantly improved in MSCs treated group, and the production of VEGF and FGF-2 was significantly higher in the MSCs group compared to groups of other kinds of cells from bone marrow. Although further basic investigations should be carried out, this clinical trial proved that MSCs hold great promise for vascular regeneration.

Both animal studies and clinical trials established the evidence that therapeutic angiogenesis with MSCs can be regarded as an effective approach for treatment of PAD. The therapy potential is based on the transplantation of MSCs, which could enhance neovascularization, thereby rescuing the ischemic tissues from degeneration. However, before we apply this approach to clinical, the therapeutic potential and scientific support of MSCs transdifferentiation need to be further investigated [51]. Future studies will be undoubtedly confident that all these basic studies and clinical trials of therapeutic neovascularization will improve outcomes in PAD.

### 3.3. MSCs for Vascular Bioengineering

Vascular grafts are increasingly needed in the clinic for coronary disease and hemodialysis [52, 53]. Unfortunately, the acute thrombosis and subsequent occlusion often lead to the failure of the transplanted synthetic acellular vascular grafts. Common models of living vascular grafts have been developed to some different types like matrix or cells alone or hybrid vascular grafts combined with cells, matrix, and soluble factors [54]. Because of their superior proliferation capacity and lower immunogenicity as an excellent cellular candidate for off-the-shelf therapy, MSCs represent a suitable alternative stem cell source [55]. MSCs have mainly three critical functions: (1) homing to the site where tissues are damaged, (2) secreting cytokines or chemokines to facilitate the migration of host cells, and (3) transdifferentiating into functional ECs [56].

In clinical application, tissue-engineered vascular grafts (TEVGs) are supposed to have mechanically durable structure and antithrombogenesis ability. Most approaches of TEVG fabrication rely on the forms of scaffold to provide mechanical integrity upon implantation to the arterial circulation.* In vivo* transplantation of acellular conduits or MSC-seeded vascular grafts as interpositional grafts suggested that MSCs could block the inflammatory responses induced by nanofibrous scaffolds [57]. The combined application of polymer grafts and MSCs for developing vascular conduits confers TEVGs with mechanical strength, good tissue compatibility, and ability of remodeling.

Genetic modification of MSCs to mimic the function of ECs has been reported. Rat BM-MSCs were transduced with eNOS and can release nitric oxide (NO) subsequently. NO is normally released by ECs to offer feasible effects of angiogenesis on the native coronary arteries, as well as improve self-patency and prevent thrombogenesis [58]. Adipose-derived cells with transfection of eNOS gene could produce significant amounts of NO, which indicate the possibility of NO production of an engineered vascular [59]. This hybrid vascular prosthesis is expected to provide a therapeutic advantage by extended production of NO from the inner surfaces.

**Figure 2:**
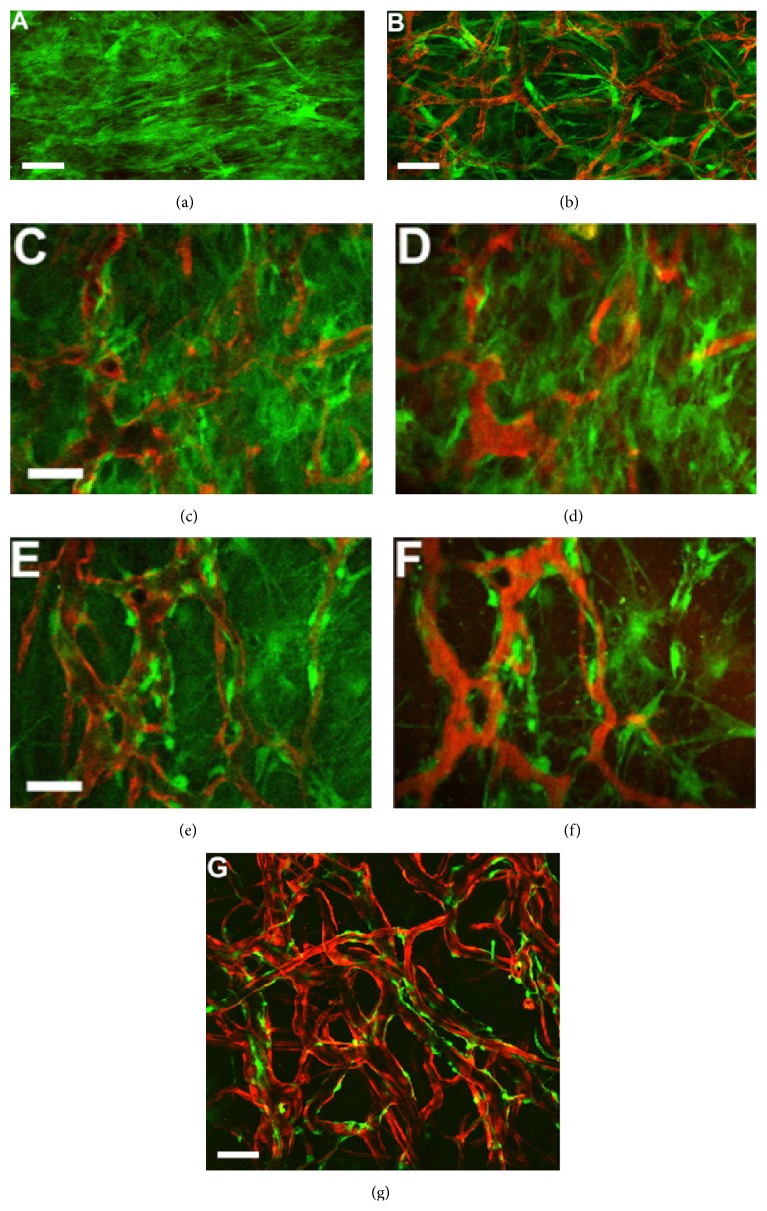
Intravital microscope analysis revealed that MSCs behaved like pericytes wrapping around the vessel in a tissue-engineered vascular model. With multiphoton laser scanning microscopy (MPLSM), images were taken at different time points. Lumen formation and blood flow in hMSCs (EGFP+) derived cells were not able to detect (a). On the contrary, implant HUVECs (DsRed+) and hMSCs (EGFP+) in mice, hMSCs (EGFP+) could be found elongate into thin slit structures and coalesced around the HUVEC-derived vessels (b)–(f). Over time, the number of interstitial hMSCs (EGFP+) was decreased, and most of them were associated with blood vessels by day 83 (g). Reprinted with permission from [60].

In addition to cells alone, to investigate spatial orientation of MSCs related to the engineered vascular, MSCs and human umbilical vein endothelial cells (HUVECs) were transduced, respectively, with retrovirus to express DsRed and EGFP [60]. They found that undifferentiated MSCs combined with HUVECs seeded on a graft are able to grow* in vivo* and function as pericytes wrapping around the endothelial tubes (Figure 2). Moreover, the angiogenic applications of MSCs are not only based on their ability to differentiate into ECs, but also on their differentiation ability toward SMCs phenotype. In response to TGF-*β*, MSCs have been revealed to transdifferentiate into SMCs by direct contact with vascular ECs, mechanical stress, and prostaglandin F_2*α*_ (PGF_2*α*_) [61, 62]. The intramuscular injection of TGF-*β*1-induced human adipose tissue-derived MSCs could improve neovascularization and blood perfusion significantly [63]. These results indicate the important role of MSCs in therapeutic angiogenesis.

## 4. Paracrine Mechanisms behind MSCs Angiogenic Potential

Despite the acute donor cell death after transplantation, both animal and clinical studies have demonstrated beneficial effects of the treatment, which indicate that stem cells perhaps act through paracrine pathways [3]. Cell-free conditioned medium from MSCs can induce angiogenesis through activation of VEGF-A expression and secretion in ECs [64]. Another experiments also revealed that conditioned medium from bone marrow or adipose tissue also has got positive effect on angiogenesis [65–67] profiting in which from MSCs' angiogenic paracrine secretion of HGF, bFGF, IGF-1, and VEGF [68]. Besides, exosomes released by MSCs may serve as an essential mediator of angiogenesis by transferring genetic materials and angiogenic molecules. Those results indicate that the paracrine cytokines and exosomes of MSCs have a complex composition rather than single molecule, all of which could participate in regenerative processes.

### 4.1. Growth Factor Production

MSCs could be recruited and mobilized to the sites of inflammation as well as injury where they can incorporate into the ischemic tissue's microenvironment. After cells transplant, the angiogenic paracrine effects of MSCs could improve tissue functions in the ischemic limb [69]. MSCs can potentiate angiogenesis via direct differentiation, cell contact interaction, or paracrine effects [70]. Angiogenic factors produced by MSCs include bFGF, VEGF, TGF-*β*, PDGF, angiopoietin-1, placental growth factor (PGF), IL-6, and monocyte chemotactic protein-1 (MCP-1), which facilitate tissue regeneration.

MSCs could stimulate angiogenesis* in vitro* and* in vivo* through secreted VEGF, MCP-1, and IL-6 into their condition medium, and these effects can be significantly inhibited by pretreatment with neutralizing antibodies against VEGF, MCP-1, and IL-6 [71]. IL-6 is an MSC-secreted cell factor displaying proangiogenic, progrowth, and prosurvival activities [72], which has potent proangiogenic and antiapoptotic activity. MCP-1 can be detected among MSC-secreted cytokines and is a critical chemoattractant for angiogenesis [67]. Last but not least, the most important proangiogenic factor VEGF has been shown possible to be expressed by MSCs as well as promote MSCs differentiation [73]. Meanwhile, VEGF has been revealed to regulate ECs migrations and differentiation and promote recruitment of ECs for angiogenesis and endothelialization in injured tissue [44]. The receptors of VEGF include VEGFR2 (*KDR/Flk1*) and tyrosine kinase receptors VEGFR1 (*Flt1*) [74]. VEGFR2 induces activation of various signaling pathways, including the activation of mitogen-activated protein kinase (MAPK), phosphoinositide-3-kinase and Akt (PI3K/AKT), Src, and Rac pathways. VEGFR2 is the main receptor involved in cell signaling for its strong tyrosine kinase activity [75].

MSCs are able to secrete a composite of angiogenic factors like HGF and stromal-cell-derived factor-1 (SDF-1), which can promote local angiogenesis [76]. SDF-1 is an inducible and constitutively expressed chemokine that can regulate multiple physiological processes, such as stimulating ECs proliferation and capillary tube formation [77]. HGF exerts its angiogenic activity through tyrosine phosphorylation of its specific receptor, c-Met, which can be found in ECs and SMCs [78]. The therapeutic efficacy of HGF has been investigated in a clinical trial [79] and shown to possess multiple effects in patients with critical limb ischemia.

MSCs have both autocrine and paracrine activities, which are involved in cell survival, organ function, and tissue angiogenesis [67, 80]. Growth factors mentioned above like VEGF, MCP-1, HGF, TGF-*β*, and IL-6 are potent angiogenic factors, which could improve angiogenesis and restoration of PAD and CVDs [81]. With these angiogenic factors, MSCs-based treatment after tissue injured could increase microvascular density and preserve organ function through increasing tissue perfusion [81]. In accordance with these observations, MSCs with various paracrine angiogenic factors are able to enhance* in vivo* angiogenesis and local blood flow recovery in the ischemic tissue.

### 4.2. Exosomes and MicroRNA Production

The angiogenic cytokines are one of the crucial factors of angiogenesis, which can support cell growth, proliferation, and communication. However, multiple studies show that the communication between MSCs and other cells is not only through cytokines but also through exerting their function via exosomes. Exosomes are small particles dynamically composed by lipids, proteins, cytoskeletal elements, molecular chaperones, and signaling molecules that can also convey biological materials to surrounding cells [82, 83]. More recently, evidences that exosomes contain genetic materials including mRNAs and microRNAs have emerged [84], and these exosomes can be functional for recipient cells. Results suggest that exosomes may serve as an essential mediator of angiogenesis by transferring genetic materials and angiogenic molecules.

Exosomes with miRNAs can be secreted by a variety of cell types such as cardiocytes, vascular cells, and MSCs in culture [85], and the altered expression of miRNAs has been correlated with CVDs. The discovery of miRNA/vesicle-mediated communication between endothelial cells and cardiovascular cells was confirmed recently [86, 87]. Moreover, miRNAs have been detected in the supernatant of human MSCs that may be associated with exosomes [88]. These exosomes were injected into a rat model of ischemia/reperfusion and revealed cardioprotective effect [89]. The result indicated that exosomes secreted by MSCs might possess therapeutic potential. Although significant progresses have been made in CVDs therapy, further investigations are needed to explore the therapeutic potency of MSCs-derived exosomes. Future clinical use of exosomes in diagnosis, monitoring disease progress, treatment, and monitoring treatment efficacy is promising.

## 5. Endothelial Differentiation of MSCs

**Figure 3:**
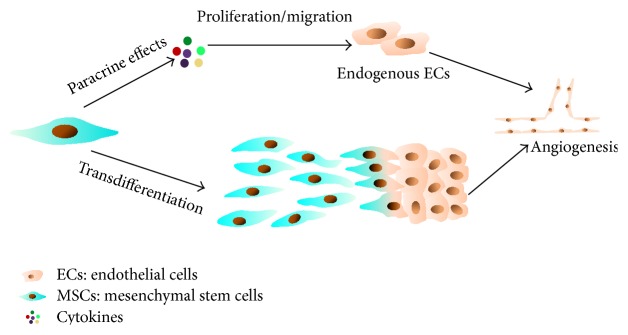
Angiogenic potency of MSCs. MSCs could promote angiogenesis either by paracrine effects or by transdifferentiation. The secretion of cytokines could enhance the proliferation and migration of endogenous endothelial cells. Moreover, MSCs may transdifferentiate into lineage with functional and phenotypic features of ECs and participate in angiogenesis directly.

Human MSCs have been confirmed to be pluripotent progenitors that can replicate as multiple types of cells including lineages with phenotypic and functional features of ECs [90]. Further* in vivo* research also showed that engrafted MSCs were positive for vWF, suggesting their transdifferentiation into ECs [91]. Since MSCs can differentiate into ECs, this means that MSCs have the ability to participate in postnatal angiogenesis and form vasculature* in vivo* [92]. Those results confirmed that MSCs could be a viable source for the* de novo* generation of ECs for therapeutic angiogenesis application (Figure 3).

Isolated MSCs were negative for typical endothelial markers like eNOS, VE-cadherin, vWF, and pericyte markers (NG2 and platelet-derived growth factor receptor *β*) [93], and those markers can be used to establish the identity of endothelial differentiation of MSCs. Moreover, MSCs isolated from human Wharton's Jelly were negative for typical endothelial markers and eNOS protein expression was increased significantly after endothelial transdifferentiation [18], which has been proved in MSCs from other sources as well. Another research interest lies in the impact of NO signaling during the process of MSCs transdifferentiation into ECs [94]. It has been revealed that NO signaling is an essential regulator of vasculogenesis in MSCs. These findings offer exciting insights for MSCs-based angiogenic therapy.

VEGF is a major factor in the process of endothelial differentiation, which can induce the endothelial differentiation of MSCs [65, 95, 96]. A study implied that VEGF induces human BM-MSCs differentiation into ECs by Rho/ROCK signaling pathway. Blocking Rho/ROCK pathway could suppress the upregulation of tube formation, migration, and proliferation of ECs [97, 98]. Moreover, PDGF could induce endothelial differentiation of MSCs [99], whereas TGF-*β* plays an important role in cell differentiation and vascular remodeling [100]. It has been revealed that TGF-*β* could increase the expression of smooth muscle *β*-actin while decreasing the expression of gelsolin according to proteomic profiling analysis [101], which will lead to more focused and in-depth research on the influences of TGF-*β* on endothelial differentiation of MSCs.

## 6. Conclusions

Over the last decade, MSCs have been proved for engineering vascular conduits or treatment of ischemic diseases. MSCs have drawn considerable attention because of their potential to differentiate into cardiovascular lineages and their promising therapeutic prospective and beneficial properties for CVDs. MSCs can be applied to a variety of clinical scenarios not only through cell-cell interactions but also through multiple paracrine factors. Therefore, understanding the intrinsic properties and associated modulations in tuning MSCs' behaviors as well as functions is indispensable for future therapeutic applications of MSCs. Clearly elucidating the mechanisms of endothelial differentiation and therapeutic angiogenesis of MSCs will offer more simple, definitive, and effective approaches for treatment of ischemic CVDs.

## Figures and Tables

**Table 1:** Completed MSC-based randomized clinical trials for ischemic heart disease therapy registered at https://clinicaltrials.gov/.

Cell	Phase	Condition	Cell delivery route	Basis of trial design	Result	Reference
Autologous BM-MSCs	II/III	AMI	Intracoronary	Repairing the damaged myocardium via paracrine signaling	Autologous BM-MSCs are safe and provide modest improvement in LVEF	[102]

Auto-hMSCs and allo-hMSCs	I/II	CILVD	Transendocardial	Prevention remodeling of the ventricle and reduction of infarct size	Alloimmune reactions of allogeneic MSCs injection are low and improved functions are observed	[103]

Autologous BM-MSCs	I/II	Heart attack	Intramyocardial	Repair and restore heart function by reducing fibrosis, neoangiogenesis, and neomyogenesis	Autologous BM-MSCs could reduce scar, enhance regional function, and improve tissue perfusion	[29]

Allogeneic BM-MSCs	I	MI	Intravenous	Transdifferentiation of MSCs into cardiomyocytes	Intravenous allogeneic hMSCs are safe in patients after AMI	[104]

Allogeneic BM- MSCs	I/II	MI	Intravenous	Transdifferentiation of MSCs into cardiomyocytes and production of new blood vessels	Intravenous infusion of allogeneic BM-MSCs is safe and well-tolerated in AMI patients	[105]

WJ-MSCs	II	STEMI	Intracoronary	Transdifferentiation of MSCs into cardiomyocytes	Intracoronary infusion of WJ-MSCs is safe and effective in patients with AMI	[106]

Autologous MSCs and BMCs	I/II	LVD	Transendocardial	Stimulation of endogenous cardiac stem cells by MSCs	Transendocardial injection with MSCs or BMCs appeared to be safe for patients with ICM and LVD	[107, 108]

AD-MSCs	II	CMI	Not special	Angiogenesis	—	—

Autologous BM-MSC	I/II	CHF	Intramyocardial	Development of new myocardium and blood vessels	Intramyocardial injections of autologous culture expanded MSCs were safe and improved myocardial function	[109, 110]

*Notes*. BM-MSCs: bone-marrow-derived human MSCs; WJ-MSCs: umbilical Wharton's Jelly-derived mesenchymal stem cell; AD-MSCs: adipose-derived mesenchymal stem cells; auto-hMSCs: autologous human mesenchymal stem cells; allo-hMSCs: allogeneic human mesenchymal stem cells; BMCs: bone marrow mononuclear cells; AMI: acute myocardial infarction; LVEF: left ventricular ejection fraction; STEMI: ST elevation myocardial Infarction; ICM: chronic ischemic cardiomyopathy; CMI: chronic myocardial ischemia; IHF: ischemic heart failure; CHF: congestive heart failure; and CILVD: chronic ischemic left ventricular dysfunction.
